# Long noncoding RNA FALEC inhibits proliferation and metastasis of tongue squamous cell carcinoma by epigenetically silencing ECM1 through EZH2

**DOI:** 10.18632/aging.102094

**Published:** 2019-07-23

**Authors:** Bo Jia, Tao Xie, Xiaoling Qiu, Xiang Sun, Jun Chen, Zhijie Huang, Xianghuai Zheng, Zhiping Wang, Jianjiang Zhao

**Affiliations:** 1 Department of Oral Surgery, Stomatological Hospital, Southern Medical University, Guangzhou 510280, People’s Republic of China; 2 Department of Stomatology, Shunde Hospital, Southern Medical University, Foshan 528300, People’s Republic of China; 3 Department of Radiation Oncology, Affiliated Cancer Hospital and Institute of Guangzhou Medical University, Guangzhou 510280, People’s Republic of China

**Keywords:** lncRNA FALEC, tongue squamous cell carcinoma, ECM1, EZH2

## Abstract

Tongue squamous cell carcinoma (TSCC), the most common epithelial cancer identified in the oral cavity, has become one of the most common malignancies across the developing countries. Increasing evidence indicates that long non-coding RNAs (lncRNAs) serve as important regulators in cancer biology. The focally amplified long non-coding RNA in epithelial cancer (FALEC) was found downregulated in the tissues of tongue squamous cell carcinoma (TSCC) and was predicted to present a good prognosis by bioinformatics analysis. Experiments indicated that FALEC knockdown significantly increased the proliferation and migration of TSCC cells both *in vitro* and *in vivo*; however, FALEC overexpression repressed these malignant behaviors. RNA pull-down and RNA immunoprecipitation demonstrated that FALEC could recruit enhancer of zeste homolog 2 (EZH2) at the promoter regions of extracellular matrix protein 1 (ECM1), epigenetically repressing ECM1 expression. The data revealed that FALEC acted as a tumor suppressor in TSCC and may aid in developing a novel potential therapeutic strategy against TSCC.

## INTRODUCTION

Oral carcinoma, the most common epithelial cancer identified in the oral cavity, has become one of the most frequently occurring malignancies across the developing countries. Approximately 25 to 40 % of oral squamous cells carcinomas were diagnosed as tongue squamous cells carcinomas (TSCC) [1–3]. Despite therapeutic management, advances in surgery, chemotherapy, and radiotherapy, the prognosis of TSCC patients has not improved significantly over the past decades. Even after proper treatment when detected early, there is a high recurrence risk or lymph node metastasis, leading to an unsatisfactory 5-year overall survival rate [4, 5]. Therefore, it is critical to further understand the specific mechanism underlying the initiation and progression of TSCC.

Long non-coding RNAs (lncRNAs) represent a novel class of RNA transcripts longer than 200 nucleotides without a protein coding attribute. LncRNAs have been shown to play crucial roles in important biological processes, such as epigenetic regulation, genomic imprinting, X-chromatin remodeling, mRNA splicing and degradation, and regulation of gene expression [6]. LncRNAs can regulate gene expression and protein synthesis by cis-regulation (< 1 Mbps from the transcribed lncRNA) or trans-regulation (> 1 Mbps from transcribed lncRNA, even on other chromosomes).

To date, several dysregulated lncRNAs have been implicated in tumorigenesis and tumor progression in various cancers, including TSCC. For example, lncRNA HOTTIP was overexpressed in TSCC patients and predicted a poor clinical outcome [7]. Decreased expression of LncRNA MEG3 was identified as an independent prognostic factor associated with poor clinical survival in TSCC patients. MEG3 inhibited cell proliferation and cell cycle progression and promoted apoptosis through activation of p53 [8]. Upregulation of MALAT1 and AFAP1-AS1 in TSCC cell lines affected cell growth, invasion, and migration abilities via Wnt signal pathway [9, 10]. Moreover, LncRNA KCNQ1OT1 regulated cell proliferation and cancer progression in TSCC via competing endogenous RNA (ceRNA) regulation [11].

Recently, focally amplified long non-coding RNA in epithelial cancer (FALEC), a novel long non-coding RNA located in focal amplicon on chromosome 1q21.2, was reported to be aberrantly expressed in a wide range of human malignances, such as ovarian cancer and prostate cancer [12, 13]. However, the expression pattern and biological functions of FALEC in oral tongue carcinoma remain to be understood.

In the present study, we observed significant downregulation of FALEC in TSCC tissues compared to tumor adjacent normal samples. The FALEC levels were inversely correlated with tumor progression and clinical outcome. Moreover, FALEC enhanced EZH2 binding to the promoter of ECM1, and inhibited ECM1 expression via mediating H3K27me3 trimethylation, thus inhibiting cell progression and migration.

## RESULTS

### FALEC is markedly decreased in TSCC and predicts a good prognosis

**Figure 1:**
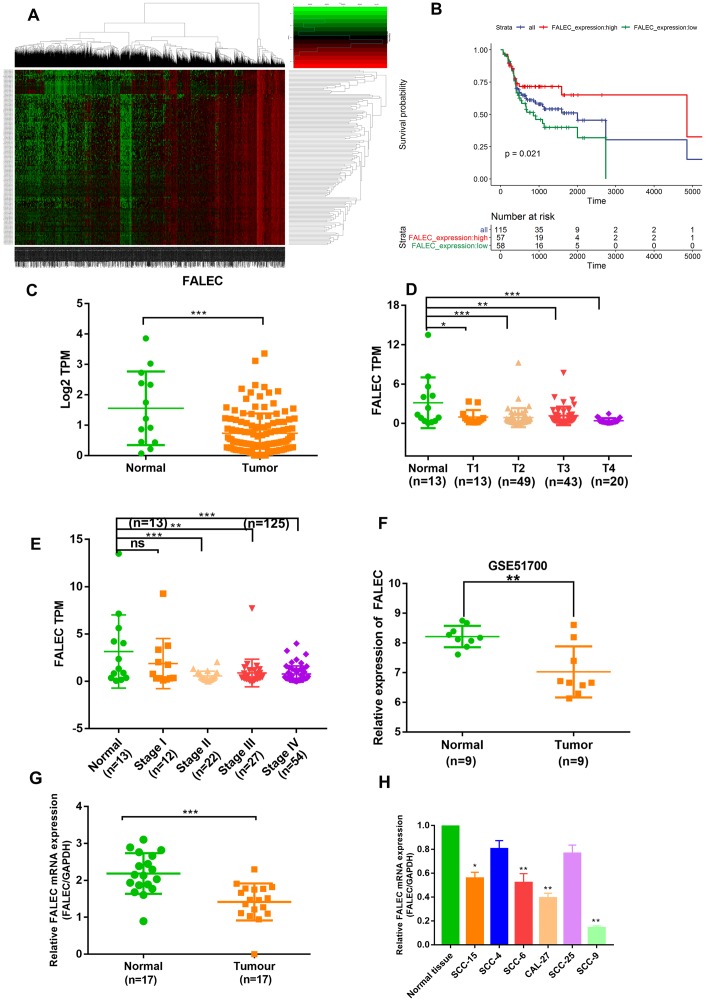
**FALEC is markedly downregulated in TSCC and predicts good prognosis.** (**A**) Hierarchical clustering analysis of lncRNAs that were differentially expressed (fold change > 2; *p* < 0.05) in tongue squamous cell carcinoma (TSCC) and normal tissues in TCGA cohort. (**B**) Kaplan-Meier curves for OS of TSCC patients with high vs. low expression of FALEC in TCGA cohort. (**C**) The Sequencing result of FALEC expression in TSCC and normal tissues in TCGA cohort. (**D**) The Sequencing result of FALEC in TSCC tissues of different T classification and normal tissues. (**E**) The Sequencing result of FALEC in TSCC tissues of different Stage and normal tissues. (**F**) FALEC expression was analyzed in GSE51700. (**G**) FALEC expression was detected in TSCC tissues and paired normal adjacent tissues. (**H**) Relative FALEC expression in cell lines and normal tissues. *P < 0.05, **P < 0.01, ***P < 0.001.

In order to explore lncRNAs involved in the progression of TSCC, we analyzed the lncRNA expression profile in pre-therapy biopsies of TSCC samples and normal samples from TCGA datasets. A mound of aberrant lncRNAs with statistically significant difference was found (Figure 1A). Kaplan-Meier survival analysis of different lncRNAs was conducted. The results showed that FALEC expression was lower in tumor tissues than in normal tissues and associated with positive prognosis in the TCGA datasets (Figure 1B–1E). Furthermore, the univariate analysis between FALEC expression levels in tongue squamous cell carcinoma (TSCC) tissues and clinicopathologic characteristics were determined. As shown in Supplementary Table 4, FALEC was significantly correlated with T classification (P < 0.05). In addition, FALEC expression was downregulated in TSCC tissues compared with both adjacent normal tissues in GEO51700 and fresh frozen tissues (Figure 1F and 1G). The expression of FALEC in normal tongue tissue was higher than TSCC cell lines (SCC-15, SCC-4, SCC-6, SCC-27, SCC-25, SCC-9) as detected by RT-qPCR (Figure 1H). These data indicate that FALEC was significantly downregulated in TSCC and may be a marker for good prognosis in TSCC patients.

### FALEC knockdown markedly promotes the proliferation and migration of TSCC cells

**Figure 2:**
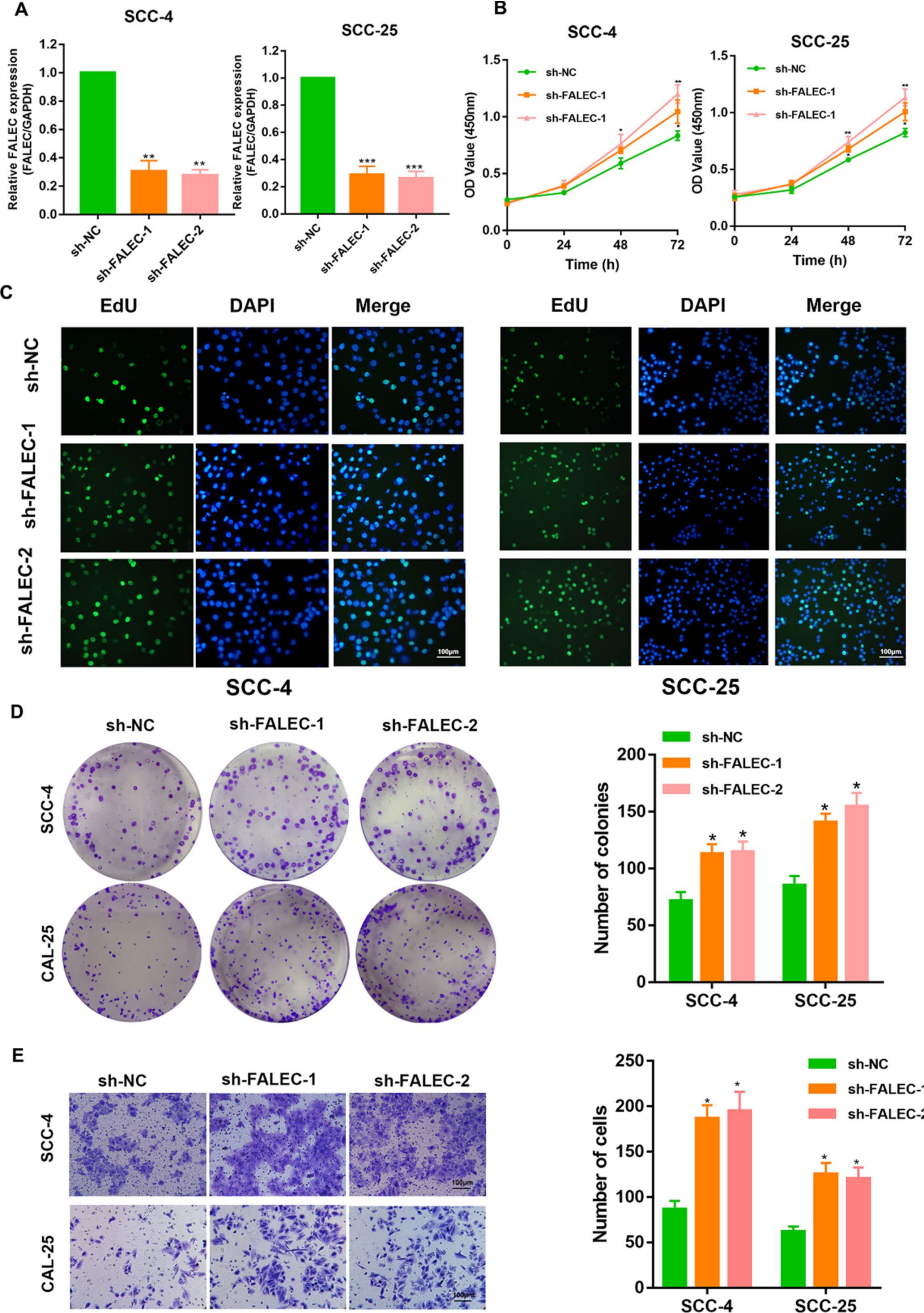
**FALEC silence promotes the proliferation and migration of TSCC cells.** (**A**) RT-qPCR analysis of FALEC was determined in FALEC-silenced and control SCC-4 and SCC-25 cell lines. (**B**) CCK-8 assays were used to determine the viability of FALEC-silenced TSCC cell lines. (**C**) EdU assays were performed to determine the proliferation of TSCC cell lines when FALEC was knockdown. (**D**) Colony formation assays were used to determine the colony-forming ability of FALEC-silenced cells. Representative images (left) and average number of colonies (right) are shown. (**E**) Transwell assays showed that FALEC silencing promoted TSCC cells migration. Representative images (left) and average number of cells (right) are shown. Data are shown as means ± SD. *p < 0.05, **p < 0.01, ***p < 0.001.

To investigate the role of FALEC in TSCC, FALEC knockdown TSCC cells were developed through two independent short hairpin RNAs (shRNAs) (Figure 2A). CCK-8 assays revealed that FALEC knockdown significantly increased cell viability in both SCC-25 and SCC-4 cell lines compared to the control cells. EdU cell proliferation assays showed that silencing of FALEC dramatically promoted the proliferation of TSCC cells (Figure 2C). Additionally, knockdown of FALEC elicited enhanced colony formation (Figure 2D). Transwell assays as well as wound healing assays showed that knockdown of FALEC dramatically increased the migration of cells (Figure 2E and Supplementary Figure 1A–1B).

### FALEC overexpression inhibits the proliferation and migration of TSCC cells *in vitro*

**Figure 3:**
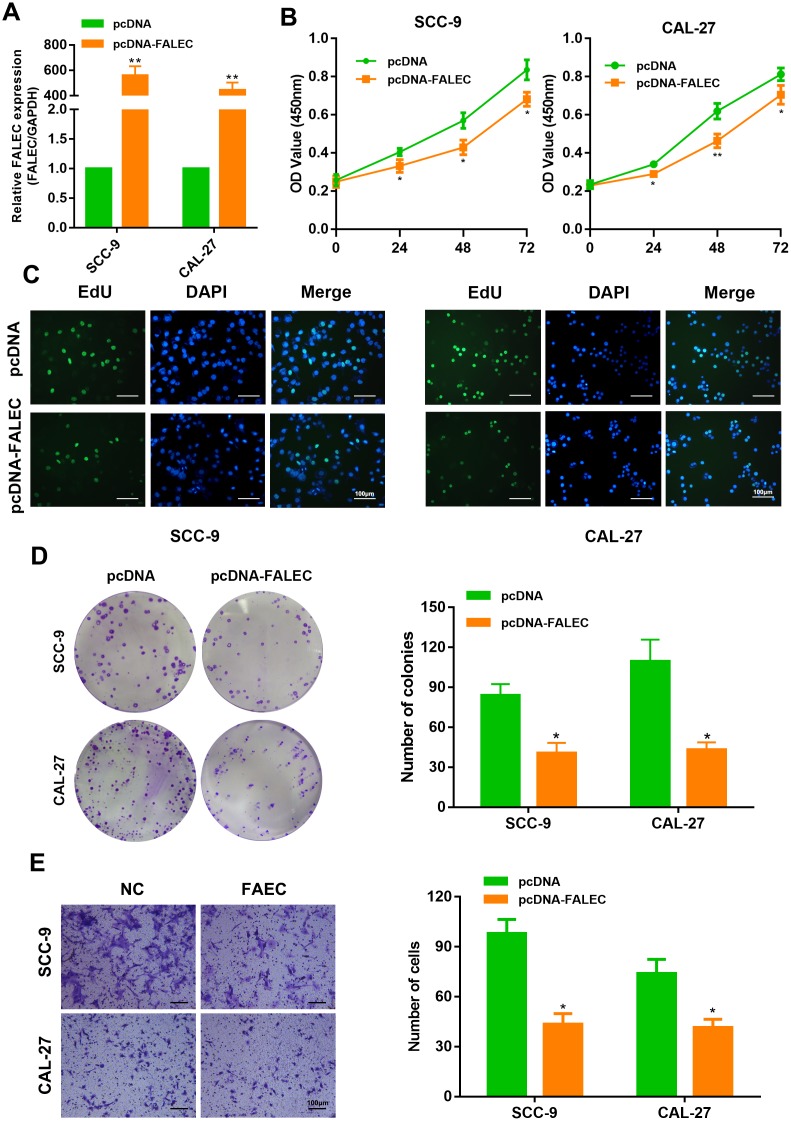
**FALEC overexpression inhibits the proliferation and migration of TSCC cells.** (**A**) RT-qPCR analysis of FALEC was determined in FALEC-overexpression and control TSCC cell lines. (**B**) CCK-8 assays were used to determine the viability of FALEC-overexpressed TSCC cell lines. (**C**) EdU assays was performed to the proliferation of cell lines when FALEC was overexpressed. (**D**) Colony formation assays were used to determine the colony-forming ability of FALEC-overexpressed cells. Representative images (left) and average number of colonies (right) are shown. (**E**) Transwell assays showed that FALEC overexpression suppressed TSCC cell migration. Representative images (left) and average number of cells (right) are shown. Data are shown as means ± SD. *p < 0.05, **p < 0.01.

We examined the effect of ectopic expression of FALEC in TSCC cells. SCC-9 and CAL-27 were selected and infected with plasmids overexpressing FALEC (Figure 3A). As expected, overexpression of FALEC inhibited cell viability and cell proliferation as found by CCK-8 and EdU assays (Figure 3B and 3C). FALEC-overexpressing cells also reduced clone numbers compared to control groups (Figure 3D). Furthermore, enforced expression of FALEC resulted in a dramatic decrease in migration ability of SCC-9 and CAL-27 as illustrated by transwell assays and wound healing assays (Figure 3E and Supplementary Figure 1C), indicating that overexpression of FALEC could restrain TSCC cell proliferation and migration.

### Overexpression of FALEC inhibits TSCC cells tumorigenesis *in vivo*

**Figure 4:**
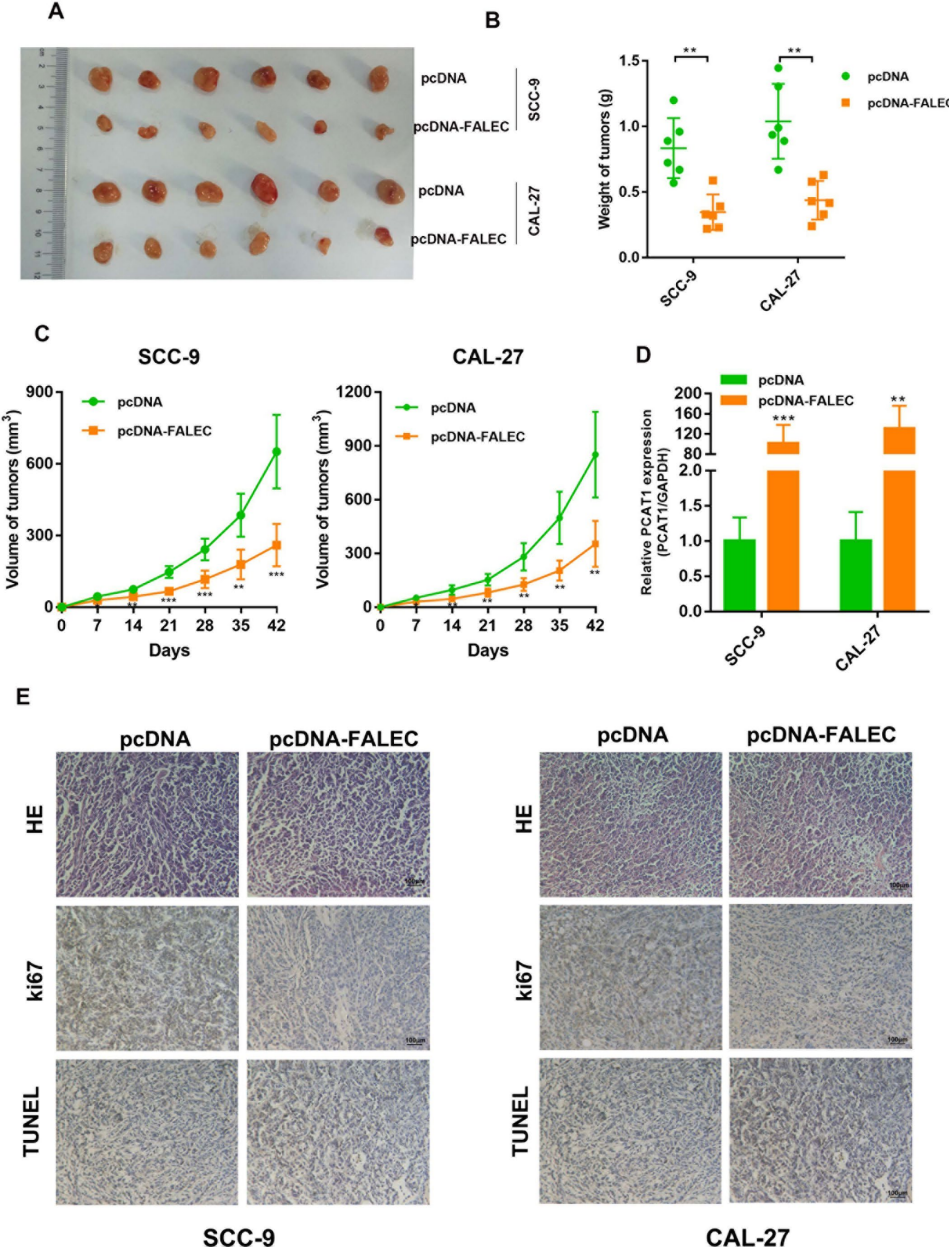
**Overexpression of FALEC significantly reduces tumor growth *in vivo*.** (**A**) Tumors removed from the Nude mice 7 weeks after injection of SCC-9 and CAL-27 cells stably transfected with pcDNA-FALEC or pcDNA, respectively. (**B**) Average weight of tumors derived from each group. (**C**) The tumor growth of FALEC-overexpression and control cells grafted mice were measured every 7 days, tumor growth curve was calculated. Representative images of tumors of each group (n = 6). (**D**)The expression of FALEC in grafted tumor tissues was analyzed by RT-qPCR. (**E**) Representative images of IHC staining of the grafted tumor. Data are shown as means ± SD. *p< 0.05, **p < 0.01***p < 0.001.

To investigate the effect of FALEC on the TSCC cell tumorigenesis *in vivo*, SCC-9 and CAL-27 cells overexpressing FALEC were subcutaneously transplanted into nude mice. As shown in Figure 4A–4C, a difference in tumor growth was observed between cells overexpressing FALEC and controls. The expression of FALEC in grafted tumor tissues was also confirmed by RT-qPCR. Moreover, immunohistochemistry (IHC) analysis of xenografted tumors showed that SCC-9 and CAL-27 cells with FALEC overexpression had significantly low cell proliferation rate (Ki-67) and high rate of apoptosis (TUNEL). Therefore, the data confirmed that FALEC suppressed the TSCC cell tumorigenesis *in vivo*.

### ECM1 is highly expressed in TSCC and negatively controlled by FALEC

**Figure 5:**
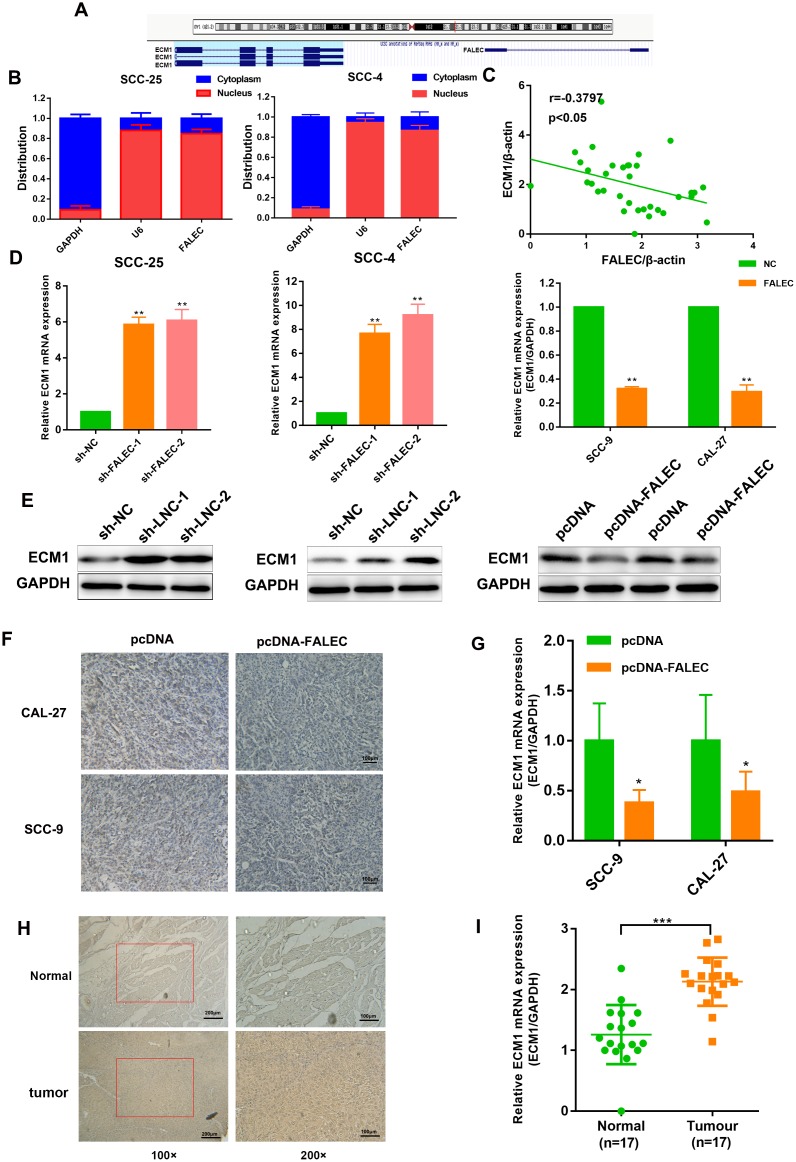
**ECM1 is highly expressed in TSCC and negatively controlled by FALEC.** (**A**) Human FALEC locus was shown using UCSC Genome Browser. There is a divergent mRNA in near from FALEC locus (**B**) RT-qPCR analysis of FALEC in the subcellular fractions of SCC-25 and SCC-4 cells. U6 and GAPDH acted as nuclear and cytoplasmic markers, respectively (n=3). (**C**) Correlation of FALEC expression and ECM1 expression in primary TSCC samples and normal samples. (**D**) RT-qPCR analysis of ECM1 in FALEC overexpressing or knockdown TSCC cell lines. (**E**) Western blotting of ECM1 in FALEC overexpressing or knockdown TSCC cell lines. (**F**) Representative immunostaining of ECM1 in FALEC overexpressing and control xenografted tumors. (**G**) RT-qPCR analysis of ECM1 in FALEC overexpressing and control xenografted tumors. (**H**) Representative immunostaining of ECM1 in TSCC tissues and adjacent normal tissues. (**I**) RT-qPCR analysis of ECM1 in TSCC tissues and adjacent normal tissues. Data are shown as means ± SD. *p < 0.05, **p < 0.01, ***p < 0.001.

LncRNAs could act as local regulators to regulate the expression of its nearby genes, named regulation “in cis” [14], inspiring us to investigate whether FALEC could regulate its neighboring genes. According to bioinformatics and gene sequence analysis, Extracellular matrix protein 1 (ECM1), a widely reported oncogene involved in the progression and metastasis of many tumors, located upstream of FALEC (Figure 5A). The subcellular distribution assays revealed that FALEC was predominately located in the nucleus (Figure 5B), suggesting its role as a intranuclear regulator. Pearson’s correlation showed that the levels of FALEC were negatively correlated with that of ECM1 in primary TSCC samples and normal samples (Figure 5C). RT-qPCR and western blot analysis indicate that overexpression of FALEC resulted in a decreased expression of ECM1, while, knockdown of FALEC increased ECM1 levels (Figure 5D and 5E). In addition, IHC as well as RT-qPCR results of ECM1 expression derived from xenografted tumors showed the same trend (Figure 5F and 5G). Moreover, an increased positive rate of ECM1 was observed in TSCC tissues rather than adjacent tissues by IHC, which was additionally verified in paired cancer adjacent tissues from 17 patients by RT-qPCR (Figure 5H and 5I). These data suggested ECM1 was negatively regulated by FALEC.

**Figure 6:**
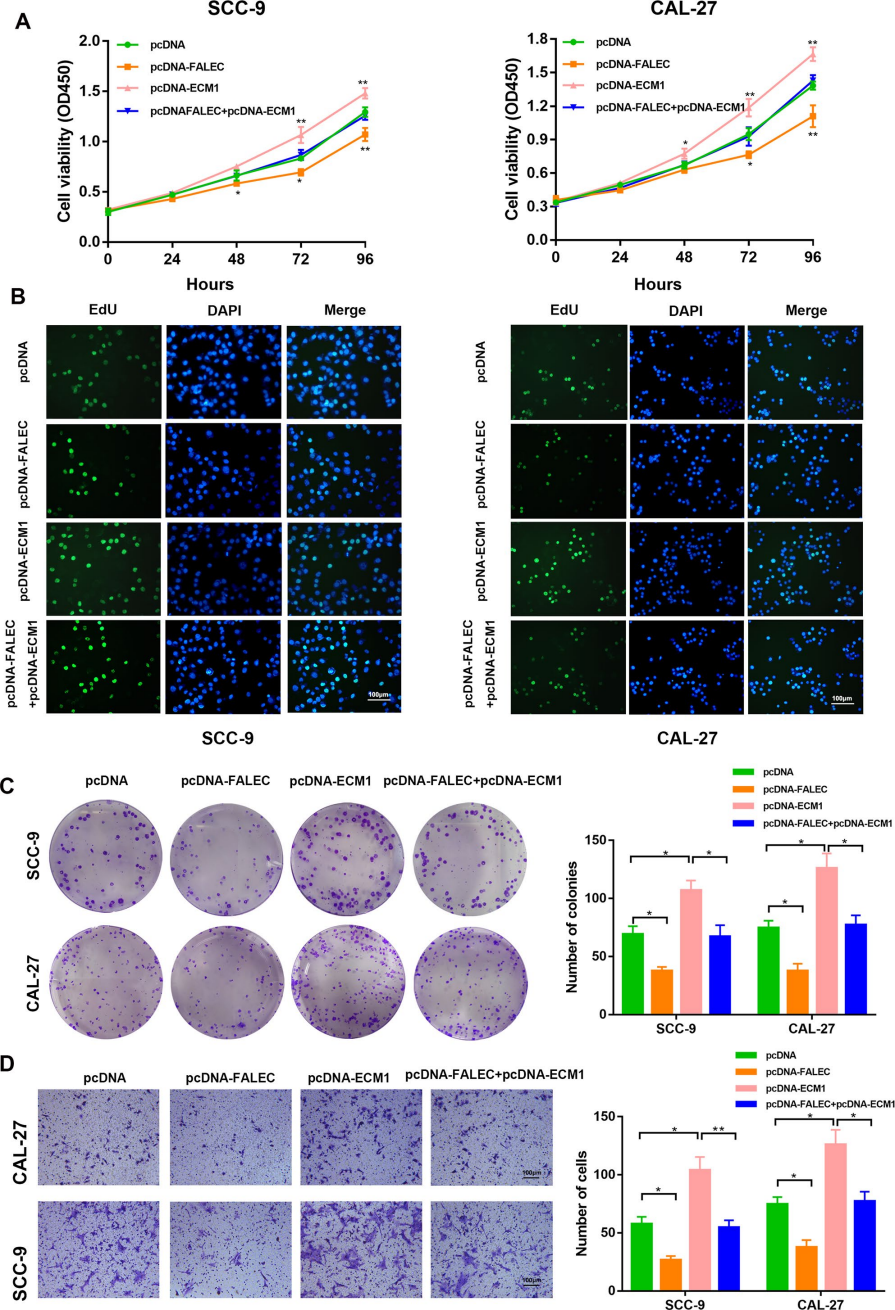
**FALEC suppress TSCC progression by downregulating ECM1.** SCC-9 and CAL-27 cells transfected with pcDNA/pcDNA-FALEC/pcDNA-ECM1 and cells transfected with pcDNA-FALEC followed by transfection with pcDNA-ECM1. After transfection, the cells were analyzed by CCK-8 assays (**A**), EdU assays (**B**), Colony formation assays (**C**) and transwell assays (**D**). Data are shown as means ± SD. *p < 0.05, **p < 0.01, ***p < 0.001.

Next, we determined the role of ECM1 in FALEC-induced suppression of TSCC cells. ECM1 upregulation significantly promoted the proliferation of TSCC cells and enhanced their migration *in vitro*. Additionally, ECM1 overexpression also rescued the repressive roles of FALEC on the malignant behaviors of TSCC cells, as illustrated by CCK-8, EdU cell proliferation assays, colony formation assays and transwell assays (Figure 6A–6D).

### FALEC binds with EZH2 to epigenetically silence ECM1, inhibiting cell proliferation and migration in TSCC cell lines.

**Figure 7:**
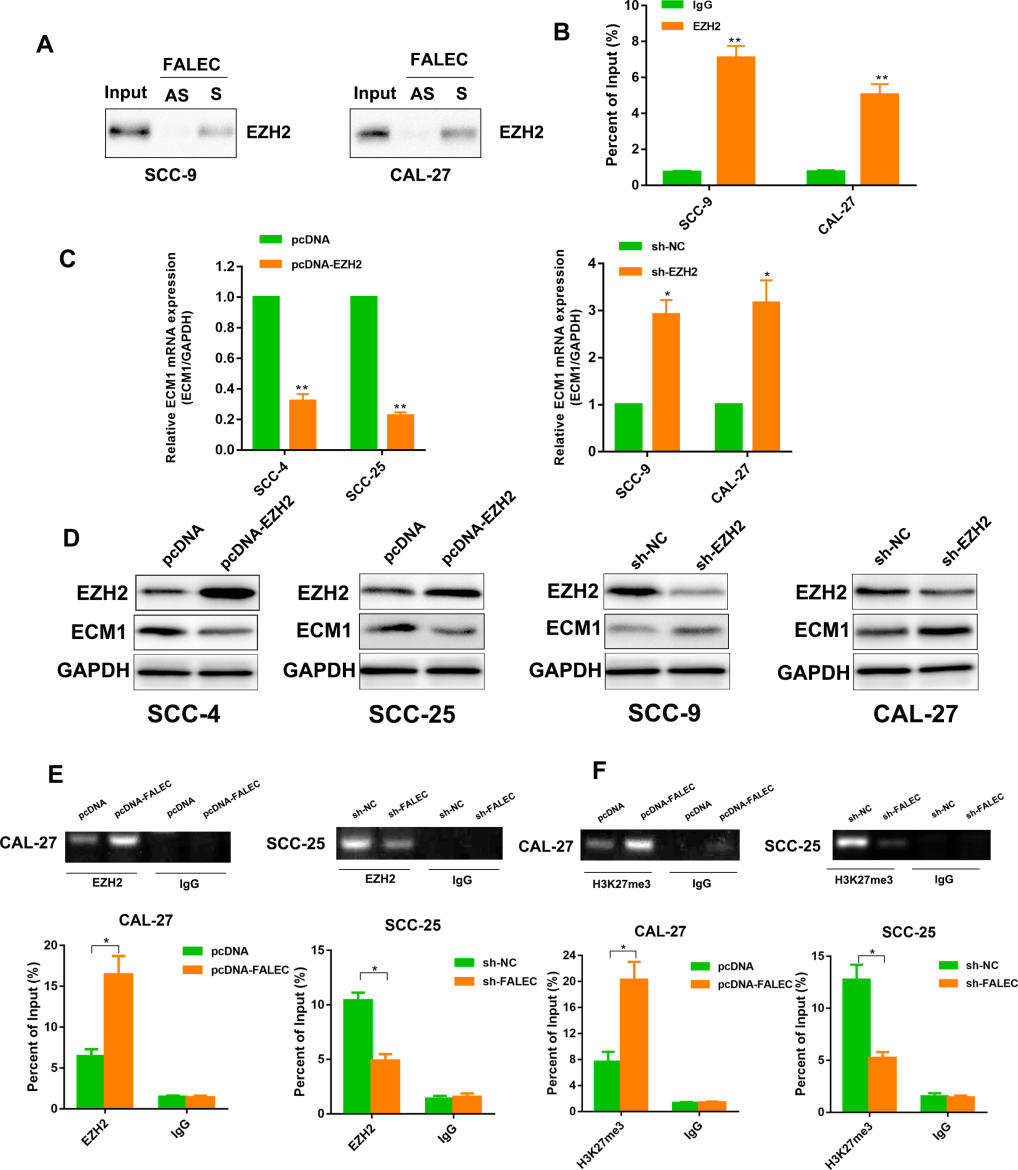
**FALEC Binds with EZH2 to Epigenetically Silence ECM1, Inhibiting Cell Proliferation and Migration in TSCC Cell Lines.** (**A**) Western Blotting of proteins from antisense FALEC and sense FALEC pull-down assays (n=3). (**B**) RNA immunoprecipitation (RIP) experiments were performed using the EZH2 antibody, and specific primers were used to detect FALEC (n=3). (**C**) ECM1 level was detected by RT-qPCR in the TSCC cell lines after overexpression or knockdown of EZH2. (**D**) ECM1 protein level was detected by western blotting in the TSCC cell lines after overexpression or knockdown of EZH2. (**E**, **F**) chromatin immunoprecipitation (ChIP) assays of EZH2 and H3K27me3 of the promoter region of the ECM1 locus after FALEC overexpression or knockdown. qPCR was performed to quantify the ChIP assays products. Enrichment was quantified relative to the input controls. IgG antibodies were used as a negative control. Data are shown as means ± SD. *p < 0.05, **p < 0.01, ***p < 0.001.

Recent studies demonstrated that lncRNAs could modulate gene silencing in cooperation with chromatin modifying enzymes, including Polycomb repressive complex 2 (PRC2). EZH2 was the catalytic subunit of the PRC2, reported to epigenetically repress transcription of specific genes [15]. To investigate whether FALEC could mediate its regulation via a similar mechanism, RNA pull-down assays as well as RNA immunoprecipitation (RIP) with antibodies against EZH2 in TSCC cells were performed. The interaction specificity between FALEC and EZH2 was confirmed (Figure 7A and 7B). Next, results of RT-qPCR and western blot confirmed the downregulation of ECM1 when EZH2 was overexpressed, in contrast, both mRNA and protein levels of ECM1 were downregulated when EZH2 was knocked out (Figure 7C and 7D). To further determine FALEC inhibited transcription through recruiting EZH2 to the promoter of ECM1, chromatin immunoprecipitation (ChIP) analysis was carried out. Results revealed that EZH2 bound to the promoter of ECM1 induced H3K27me3 modifications. FALEC knockdown decreased EZH2’ binding as compared to the control group, whereas, the recruitment of EZH2 and H3K27me3 levels in the promoter of ECM1 increased when FALEC was overexpressed (Figure 7E and 7F). These data confirmed that EZH2 could directly bind to the promoter of ECM1 mediating H3K27me3 methylation with the help of FALEC.

## DISCUSSION

Oral squamous cells carcinoma is one of the most common cancers found worldwide, which imparts a huge burden on the society due to its increasing recurrence rate and prevalence of risk factors [1, 16]. For a better understanding of the development and progress of oral squamous cells carcinoma it is essential to investigate the molecular mechanisms underlying the initiation and progression of oral squamous cells carcinoma and to explore the sensitive diagnostic biomarkers and effective therapeutic targets.

In the past decades, fresh evidence have indicated that lncRNAs were involved in different biological functions in diverse human diseases, particularly in human cancer initiation and progression and metastatic spread, including TSCC [17–20]. However, their biological functions and mechanisms of action are still not completely understood.

High-throughput technologies yielding massive data of tumor profiles, have been employed as an advanced approach for cancer study. The application of bioinformatic analysis in TCGA datasets have uncovered specific genes whose aberrant expression may be critical in cancer initiation and progression. In order to identify lncRNAs that might be implicated in TSCC, lncRNA expression data of TCSS patients pre-therapy was collected from TCGA. A novel lncRNA FALEC was markedly downregulated in TSCC tissues, further validated by clinical TSCC samples in our cohort. In addition, elevated expression of FALEC was associated with a good prognosis. This finding elucidated the fact that FALEC might play a significant role in TSCC malignancy. Furthermore, FALEC knockdown significantly increased the proliferation and migration of TSCC cells both *in vitro* and *in vivo*, on the contrary, FALEC overexpression repressed these malignant behaviors. These studies indicated that FALEC might act as an anti-oncogene in the tumorigenesis of TSCC.

Since lncRNAs often exert their effects and regulate their nearby gene in cis-regulation, we targeted the FALEC locus using UCSC Genome Browser, and found ECM1, separated by a distance of<1 kb, which is a divergent mRNA of FALEC. ECM1 is upregulated in a variety of malignant epithelial tumors, including invasive breast ductal carcinoma, colorectal cancer, esophageal squamous carcinoma and gastric cancer, and functions as a oncogene in tumor development and prognosis [21–23]. ECM1 promotes tumor metastasis through epithelial–mesenchymal transition (EMT) progression, L Gan demonstrated ECM1 induced SOX2 expression via direct interaction with integrin β4 (ITGB4) in gastric cancer and thus altering gene expression of EMT factors [24]. ECM1 also proved to be associated with breast cancer. By increasing the association between β-catenin and the MUC1 cytoplasmic tail, ECM1 regulated the EMT progression and cancer stem cell proliferation [25]. In addition, ECM1 promoted aggressive phenotype of breast cancer by recruitment of MSN to promote its activation for invadopodia formation, which was closely correlated with breast cancer invasion [26]. Nevertheless, the function of ECM1 in the tumorigenesis of TSCC remains unknown. Our data revealed that the levels of FALEC were negatively correlated with that of ECM1 by Pearson’s correlation. Furthermore, overexpression of FALEC resulted in a decreased expression of ECM1, while knockdown of FALEC increased ECM1 levels *in vitro* and *in vivo*. To determine whether FALEC regulated TSCC cell growth and metastasis by blocking ECM1 expression, rescue assays were performed. The results showed that ECM1 could partly reverse FALEC-mediated repressive roles on the malignant behaviors of TSCC cells.

Recent studies demonstrated that lncRNAs could modulate gene silencing in cooperation with enzymes involved in histone protein modification or transcription factors to specific genomic loci [27]. PRC2, which is composed of EZH2, SUZ12 and EED, has histone methyltransferase activity and primarily H3K27me3, thus epigenetically modulating gene expression [28]. EZH2 is the catalytic subunit of the PRC2, reported to epigenetically repress transcription of specific genes. For instance, lnc-LBCS could dramatically attenuate bladder cancer initiation and chemo-resistance via guiding hnRNPK-EZH2 complex to the SOX2 promoter and mediating histone H3 lysine 27 trimethylation (H3K27me3) [29]. Similarly, PVT1 could bind epigenetic modification complexes (PRC2) for histone methylation in the promoter of ANGPTL4, and thus, promote the malignancy of Cholangiocarcinoma [30]. Consistent with previous research, FALEC could mediate its regulation via a similar mechanism. RNA pull-down assay, RIP as well as ChIP assay with antibodies against EZH2 in TSCC cells confirmed FALEC inhibited ECM1 transcription through recruiting EZH2 to its promoter and induced H3K27me3 Modifications.

## MATERIALS AND METHODS

### Human tissue samples

A total of 17 pairs of fresh TSCC (TC) tissues and normal adjacent tissues were obtained from patients, who were diagnosed and underwent surgery in Stomatological Hospital, Southern Medical University, and stored at -80°C. This study was approved by the hospital institutional review board and written informed consent was obtained from all the patients. All the protocols were reviewed by the Joint Ethics Committee of the Southern Medical University Health Authority and performed following national guidelines.

### Microarray analysis

To gain insight into the potential FALEC function in TSCC, the cancer Genome Atlas (TCGA) HNSCC cohort sequence and clinical data were downloaded from the TCGA (TCGA, https://cancergenome.nih.gov/). TSCC-related LncRNA expression profile datasets GSE51700, were downloaded from the Gene Expression Omnibus (GEO, http://www.ncbi.nlm.nih.gov/geo/). The TCGA HNSCC cohort consisted of 545 samples with RNA-seq data, including 138 TSCC samples. Limited clinical information was available for these TCGA samples; thus, only the variables of survival data and tumor-node-metastases (TNM) stage were used for downstream analysis. Patients with unknown TNM stage and survival messages were excluded from further analysis, leaving a cohort of 128 TCGA TSCC samples (115 tumor tissue samples and 13 adjacent normal samples). The differential analysis was performed by edge R and LIMMA package in R (version 3.5.1) Foldchange > 2 and FDR < 0.05 were used as cut-off values.

### Quantitative real-time PCR analysis

Total RNA was extracted from tissues or cells using TRIzol reagent (Invitrogen, CA, USA) according to the manufacturer’s instructions. Total RNAs were reverse transcribed into the cDNA using a PrimeScript RT reagent kit (TaKaRa, Tokyo, Japan). RT-qPCR analyses were performed using SYBR Green Master Mix (TaKaRa, Tokyo, Japan). This process was performed using ABI Prism 7500 HT sequence detection system (Applied Biosystems, Foster City, CA) under the following conditions: 3 min at 95 °C, 15 s at 95 °C and 30 s at 60 °C for 40 cycles. The results were normalized with the expression of glyceraldehyde-3-phosphate dehydrogenase (GAPDH). All the sequences of lncRNA primers used for RT-qPCR in this study are listed in Supplementary Table 1.

### Cell culture

Human TSCC cell lines (SCC-9, CAL-27, SCC-25, SCC-4, SCC-6, SCC-15) were purchased from American Type Culture Collection (ATCC, Manassas, USA). Cells were incubated at 37 °C in a humidified atmosphere of 5 % CO_2_, maintained in Dulbecco’s modified Eagle’s medium (DMEM, Gibco, CA, USA) supplemented with 10 % fetal bovine serum (FBS, Gibco, CA, USA), 100 U/mL penicillin, and 100 mg/mL streptomycin.

### Transfection of cell lines

The full length FALEC, ECM1, and EZH2 cDNAs were synthesized by RiboBio (Guangzhou, China) and cloned into pcDNA3.1(Invitrogen), named pcDNA-FALEC, pcDNA-ECM1 and pcDNA-EZH2, which were confirmed by sequencing (Ruibiotech, Guangzhou, China). An empty vector was used as a negative control. For depletion, FALEC-targeting short-hairpin (sh)RNA and EZH2 targeting short-hairpin (sh)RNA were introduced and cloned into pLKO.1-puro vector (Sigma-Aldrich). Sequences of shRNA against specific target were listed in Supplementary Table 2. Cells were seeded in 6-well plates and incubated in DMEM with 10 % FBS for 24 h before transfection. Cell transfection was performed with the help of Lipofectamine 3000 (Invitrogen, CA, USA) following the manufacturer’s protocols.

### Cell proliferation assays

Cell proliferation was determined using cell counting kit-8 (CCK-8) assays. SCC-9 and CAL-27 cells were seeded in 96-well plates (5 × 10^3^ cells/well) and the absorption of the cells was measured using a CCK-8 kit (Dojindo, Japan) following the manufacturer’s protocols at different time points. Wells were measured in triplicates for the different treatment groups.

### Colony formation assays

For the colony formation assays, 1 × 10^**3**^ transfected cells were inoculated into 6-well plates with 2 mL DMEM supplemented with 10 % FBS for 14 days, with the replacement of medium every 3 days. The resulting colonies were fixed with 4 % paraformaldehyde and stained with Giemsa (Sigma-Aldrich, USA) for 40 min. Colony formation was determined as the quantity of visibly stained colonies. Wells were measured in triplicates for each group.

### Transwell assays

Cell migration ability was measured using transwell chambers (24-well insert, 8 μm, Millipore, MA, USA). To the upper chamber of a transwell insert, 2 × 10^4^ cells in medium with 1 %FBS were added, while DMEM containing 10% FBS was added to the lower chamber. After 24 h of incubation, the number of cells passing through the bottom membrane of the chamber were dyed with methanol and 0.1 % crystal violet and counted under a microscope (Nikon, Japan). The experiment was performed in triplicates.

### EdU assays

Cell proliferation was determined using the Cell-Light EdU DNA Cell Proliferation Kit (KeyGEN BioTECH, China). Cell lines of SCC-4, SCC-25 were seeded in 96-well plates and incubated with 50 μmol/L of EdU for 4 h at 37 °C. Further, cells were fixed with 4 % paraformaldehyde for 30 min and exposed to 1×Apollo reaction cocktail for 30 min followed by incubation with DAPI to stain the cell nuclei for 15 min. Images were captured by a fluorescence microscope (Nikon, Japan). The experiment was performed in triplicates.

### Wound-healing assays

Cell lines of SCC-4, SCC-25, SCC-9, CAL-27 were seeded at a density of 1 × 10 ^6^ in 6-well plates and cultured, respectively. Cell layers were scratched by a 200 μL tip to form wounded gaps while on reaching 90 % confluence of each plate, then cultured with medium containing 2 % FBS for 24 h. The wounded gaps were photographed and analyzed. The percentage of wound healing was calculated by the equation; (percentage of wound healing) = average of [(gap area: 0 hours) - (gap area: 48 hours)/ (gap area: 0 hours)].

### *In vivo* tumor formation assays

Twenty-four 8-week-old male mice were bought from Guangdong Medical Laboratory Animal Center, and kept under specific pathogen-free conditions. Mice were divided into 4 groups at random and inoculated subcutaneously on the right flank with 2 × 10^6^ cells (n = 6 per experimental group), respectively. The tumor growth was observed and the tumor volume was measured every week. The animals were sacrificed after 7 weeks and the tumors were excised for histopathological examination.

### Histological analysis

TSCC tissues were fixed in 4 % paraformaldehyde and then embedded in paraffin. Hematoxylin and eosin (H & E) staining was performed as previously described [31]. For immunohistochemistry analysis, Ki67 were detected in both human TSCC tissues and xenograft tumor tissues from nude mice. The aforementioned paraffin sections were deparaffinized, hydrated, antigen retrieved and blocked, and then incubated in primary antibody (Ki67, 1:500, Abcam, UK), ECM1 (1:500, Abcam, UK) overnight at 4 °C, following incubation in a solution of anti-rabbit IgG (Abcam, UK) for 15 min at room temperature and stained using a 3,3-diaminobenzidine color kit. The pictures were captured using a light microscope (Nikon, Japan). The TUNEL assays was performed using the In Situ Cell Death Detection Kit (Roche), following the manufacturer’s protocol.

### Western blotting

Total protein was extracted from cells and tissues using RIPA solution (Beyotime, China) according to the manufacturer’s instruction. Equivalent amounts of proteins from each sample were separated by 10 % SDS-PAGE, transferred to 0.22 μm PVDF membranes (Millipore, MA, USA), blocked in 5 % fat-free milk for 1 h at room temperature and incubated with specific antibodies. Primary antibodies used are as follows: ECM 1(1:1000, Abcam), EZH2 (1:1000, Cell Signaling Technology). The membranes were then incubated with HRP-conjugated IgG for 2 h at room temperature, followed by detection with enhanced chemiluminescence system. A GAPDH antibody was used as control. The experiment was performed in triplicates.

### RNA-pulldown assays

Biotin-labeled lncRNA-FALEC full-length sense and antisense were obtained by TranscriptAid T7 High Yield Transcription Kit. Biotin-labeled RNAs in refolding buffer (10mM Tris pH 7.5, 0.1 M KCl and10 mM MgCl2) were added into cell lysates and incubated for 4 h at 4 °C, followed by addition of beads. After washing thrice in the lysis buffer containing 1 mM EDTA and 0.5 mM DTT, beads were boiled and precipitated proteins were separated by SDS-PAGE and determined through western blot analysis.

### RNA immunoprecipitation (RIP) assays

A RIP assay was carried out to study whether FALEC could interact with EZH2 with a Magna RIP RNA-binding protein immunoprecipitation kit (Millipore, Billerica, MA, USA). Cells were lysed with RIP buffer (20 mM Tris pH 7.5, 150 mM NaCl, 1 mM MgCl2, 0.1 % NP40, 5 % glycerol, and 0.5 mM DTT) supplemented with RNase inhibitor; then, the cell extract was mixed with agarose beads, which had already precipitated with the protein antibodies. The retrieved proteins were detected by western blotting and the co-precipitated RNAs were detected by RT-qPCR.

### Chromatin immunoprecipitation (ChIP) assays

TSCC cells were prepared for ChIP assays using a ChIP assay kit (Millipore, MA, USA) according to the manufacturer’s instructions. Antibodies against EZH2 (1:50, Cell Signaling Technology) and H3 trimethyl Lys27 (H3K27me3) (1:50, Cell Signaling Technology) were obtained. The sequences of the ChIP primers are shown in Supplementary Table 3. The PCR products were resolved electrophoretically on a 2% agarose gel and visualized using ethidium bromide staining.

### Statistical analysis

All experimental data were analyzed using SPSS 20.0 (SPSS Inc., Chicago, USA) and GraphPad Prism 7 (GraphPad, CA, USA). Results with normal distribution were presented as mean ± SD. The statistical significance of the differences between various groups was calculated with Student’s t tests or one-way ANOVA analysis as appropriate. Pearson’s correlation and chi-square test were used to analyze the clinical variables. Spearman’s correlation analysis was performed to determine the correlation between two variables. Cumulative survival time was calculated using the Kaplan-Meier method and analyzed by the log-rank test. A *P* value of <0.05 was considered statistically significant.

## Supplementary Material

Supplementary Figure 1

Supplementary Tables
