# Supplementary material for: Long noncoding RNA FALEC inhibits proliferation and metastasis of tongue squamous cell carcinoma by epigenetically silencing ECM1 through EZH2
Source: Aging (Albany NY). 2019 Jul 23;11(14):4990–5007. doi: 10.18632/aging.102094 (PMC6682530; doi:10.18632/aging.102094)
Supplement: Supplementary Figure 1 [file aging-11-102094-s002.pdf]

SUPPLEMENTARY FIGURE

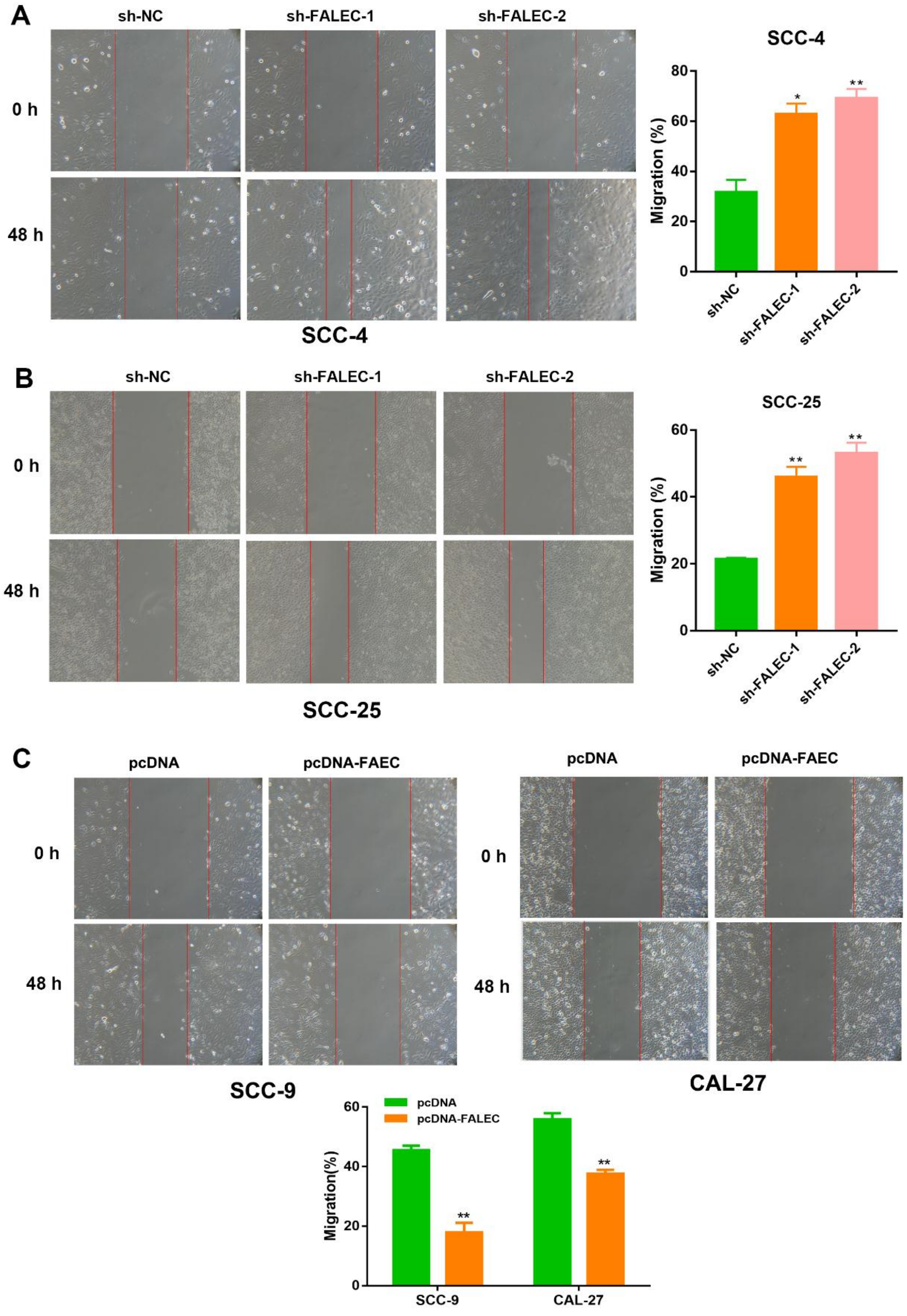

Supplementary Figure 1. (**A**, **B**) Wound-healing assay showed that FALEC knockdown promotes TSCC cell migration. Representative images (left) and average migration (right) are shown (**C**) Wound-healing assay showed that FALEC overexpression suppressed TSCC cell migration. Representative images (up) and average migration (down) are shown. Data are shown as means ± SD. *p < 0.05, **p < 0.01.
