# Supplementary material for: Long noncoding RNA FALEC inhibits proliferation and metastasis of tongue squamous cell carcinoma by epigenetically silencing ECM1 through EZH2
Source: Aging (Albany NY). 2019 Jul 23;11(14):4990–5007. doi: 10.18632/aging.102094 (PMC6682530; doi:10.18632/aging.102094)
Supplement: Supplementary Tables [file aging-11-102094-s001.pdf]

SUPPLEMENTARY TABLES

Supplementary Table 1. Sequences of PCR primers used in this study.

FALEC	Forward(5'-3')	TGGATTGTT GGGTATATTTTGGA
	Reverse(5'-3')	TGTATGAAG AGGATGCTGAAGGC
ECM1	Forward(5'-3')	GACCTATTTGGCTGTTGCTTCT
	Reverse(5'-3')	GGGGACCCACTTCCTTTTCA

Supplementary Table 2. Sequences of shRNA Against Specific Target.

sh-FALEC-1	5'-3'	GGATCACGAGGTAGGAGTTAA
sh-FALEC-2	5'-3'	CCAACAGGAGTCTAATCAA
sh-EZH2	5'-3'	CGGCTCCTCTAACCATGTTTA
sh-NC	5'-3'	TTCTCCGAACGTGTCACGT

Supplementary Table 3. Sequences of ChIP primers.

ECM1	Forward(5'-3')	AACACGAGGGTCCCAAAT
	Reverse(5'-3')	GGTCCCCAAACCAAGTCT

Supplementary Table 4. Correlation between FALEC expression and clinicopathologics of tongue cancer patients.

**Variables**	**No. of patients (n=115)**	**FALEC expression**		χ^2	**P value**	**Fisher P value**
		**High n=59(%)**	**Low n=56(%)**			
AGE(years)				3.2306	0.072	
<50	28	19(32.2)	9(16.1)			
>50	87	40(67.8)	47(83.9)			
gender				0.14684	0.702	
males	75	37(62.7)	38(67.9)			
famales	40	22(31.3)	18(31.1)			
Grade	15	10(16.9)	5(8.9)	4.9624	0.084	
G1	0	42(71.2)	36(64.3)			
G2	0	7(11.9)	15(26.8)			
G3						
T classification				9.1808	0.027	0. 027
T1	10	7(11.9)	3(5.4)			
T2	27	23(39.0)	24(42.9)			
T3	38	24(40.7)	14(25.0)			
T4	20	5(8.5)	15(26.8)			
T classification (T1+T2/T3+T4)				0.0091622	0.924	
T1+T2	57	30(50.8)	27(48.2)			
T3+T4	58	29(49.2)	29(51.8)			

Stage				0.92237	0.82	
Stage I	12	6(10.2)	6(10.7)			
Stage II	22	11(18.6)	11(19.6)			
Stage III	17	16(27.1)	11(19.6)			
Stage IV	54	26(44.1)	28(50.0)			
STAGE(I+II/III+IV)				6.24E-31	1	
Stage I+II	34	17(28.8)	17(30.4)			
Stage III+IV	81	42(71.2)	39(69.6)			
Lymph nodes metastasis				3.1042	0.078	
No	60	36(61)	24(42.9)			
Yes	55	23(39.0)	32(57.1)			
Distant metastasitc				0.00068697	0.979	0.487
No	114	59(100)	55(98.2)			
Yes	1	0(0.0)	1(1.8)			

*$p<0.05$. Pearson's Chi-squared test and Fisher's Exact Test
